# Controlling Unpleasant Thoughts: Adjustments of Cognitive Control Based on Previous-Trial Load in a Working Memory Task

**DOI:** 10.3389/fnhum.2019.00469

**Published:** 2020-01-24

**Authors:** Luiza Bonfim Pacheco, Jéssica S. Figueira, Mirtes G. Pereira, Leticia Oliveira, Isabel A. David

**Affiliations:** ^1^Department of Neurobiology, Institute of Neurobiology, Universidade Federal Fluminense, Niteroi, Brazil; ^2^Melbourne School of Psychological Sciences, The University of Melbourne, Melbourne, VIC, Australia; ^3^Physiology and Pharmacology Department, Biomedical Institute, Universidade Federal Fluminense, Niteroi, Brazil; ^4^Department of Psychology, Center for the Study of Emotion and Attention, University of Florida, Gainesville, FL, United States

**Keywords:** working memory, conflict adaptation, event-related potentials, emotion, thought control ability

## Abstract

Dynamic cognitive control adjustments are important for integrating thoughts and actions that take place during dynamic changes of environmental demands and support goal-directed behavior. We investigated, in a working memory (WM) paradigm, whether dynamic adjustments in cognitive control based on previous trial load influence the neural response to neutral or unpleasant distracters. We also investigated whether individual self-reported abilities in controlling thoughts influence this effect. Participants performed a WM change detection task with low or high WM-related cognitive demands. An unpleasant or a neutral distractive image was presented at the beginning of each trial, prior to the WM task. We tested for control adjustments that were associated with the load level of the preceding trial task (N-1) on the neural response to the subsequent distractive image. We found an effect of the prior WM task load on a parieto-occipital waveform event-related potential (ERP) that appeared between 200 and 300 ms after the neutral distracter onset. This effect was not observed for the unpleasant distracter. Individual ability for controlling thoughts may influence the effect of cognitive control adjustments on distracter processing during the unpleasant condition. These findings provide evidence that: (1) dynamic cognitive control adjustments are impaired by unpleasant distracters; and (2) the ability to control unpleasant thoughts is linked to individual differences in flexible cognitive control adjustments and shielding of WM representations from unpleasant distracters.

## Introduction

In everyday life, we must be able to flexibly integrate thought and action and keep behavior functional during dynamic changes in environmental demand. Cognitive control processes can be reactively triggered by immediate changes in task demands or deployed proactively by adjusting thoughts and actions in accordance with previously set proactive cognitive strategies that are aimed to optimize behavior (Braver, 2012). Many studies have observed proactive cognitive control adjustments experimentally by observing an increase in task performance during a current trial that follows a demanding previous trial (Gratton et al., 1992; Kerns et al., 2004; Jha and Kiyonaga, 2010). In conflict-embodied cognitive tasks, this trial-by-trial control adjustment is known as conflict adaptation (Gratton et al., 1992). The rationale here is that when participants experience an incongruent trial, where there is conflicted information between the relevant and irrelevant stimuli (e.g., the word “RED” printed in green ink in the case of a Stroop task), the participants upregulate control in order to be in a better position to handle conflict during the subsequent trial (Gratton et al., 1992; Botvinick et al., 2001; Egner, 2007; Padmala et al., 2011). This cognitive adaptation, as a function of the cognitive demands of a previous trial task, can also be observed during a working memory (WM) task (Jha and Kiyonaga, 2010; Samrani et al., 2018).

WM is responsible for keeping relevant information in mind while inhibiting intrusive irrelevant information, and therefore plays a putative role in cognitive control and task performance (Baddeley, 2012). Indeed, many studies argue that WM plays a role in control adjustments, such as conflict adaptation (Braver et al., 2007; Mansouri et al., 2007; Weldon et al., 2013; Gulbinaite et al., 2014; Kim et al., 2014; Redick, 2014). It has also been shown that individuals with high WM capacity tend to have greater success in flexibly carrying proactive control across trials and therefore adapt more efficiently to the presence of conflict, even when incongruent trials are rare within the task (Weldon et al., 2013; Gulbinaite et al., 2014).

Previously, we showed that a negative emotional state (induced by presenting distractive negative pictures) disrupts and reduces WM capacity, which was measured using the electrophysiological index denominated contralateral delay activity (CDA; Figueira et al., 2017). This finding corroborates with the extensive literature that indicates that our ability to perform goal-directed tasks can be disrupted by emotional responses to distractive affective stimuli (Vuilleumier and Schwartz, 2001; Erthal et al., 2005; Dolcos and McCarthy, 2006; Fernandes et al., 2013; Oliveira et al., 2013; Stout et al., 2013). Additionally, Figueira et al. (2017) demonstrated that the emotional state effect on WM capacity is influenced by thought control ability traits (Luciano et al., 2005). Individuals who experienced more intrusive thoughts were more susceptible to the effect of the emotional state on WM capacity (Figueira et al., 2017). Besides a high level of thought control ability being associated with higher cognitive control (Williams et al., 2010), low thought control ability is associated with measures of anxiety and also symptomatology for a number of psychological disorders (Luciano et al., 2005). This highlights the importance of understanding more about this trait and its possible clinical implications. It is important to note that the study by Figueira et al. (2017) focused on the influence of unpleasant and neutral stimuli on the ongoing WM trials. Thus, Figueira et al. (2017) investigated the effect of emotion on cognitive control processes that are reactively triggered by immediate changes in task demands. In the present study, we focused on how proactive cognitive control adjustments, based on the cognitive demands of a previous trial task, may influence the neural response to unpleasant and neutral stimuli that should not be attended by the participants (i.e., distractive stimuli; Pessoa and Pereira, 2013).

The processing of distractive stimuli presented during a goal-relevant cognitive task is likely to be influenced by the upregulation of control that is imposed by the previous trials’ cognitive demands. In other words, the neural response to distractive non-emotional information can be influenced by proactive cognitive control that acts prior to the onset of a distracter (Geng, 2014). For instance, non-emotional distracter processing is minimized when it is expected (Forster and Lavie, 2008; Braver, 2012; Grimshaw et al., 2018) or when the previous trial is demanding (Gratton et al., 1992). The processing of emotional distracters, on the other hand, might be less affected by proactive cognitive control strategies. Because of their relevance to survival, the processing of emotional-laden stimuli is prioritized and may compete with the main task for processing resources (for review, see Oliveira et al., 2013). In a Stroop-task behavioral study, Padmala et al. (2011) found that, compared to neutral images, task-irrelevant unpleasant images that were shown between trials significantly reduced conflict adaptation effects. They interpreted these findings in terms of shared resources between proactive control mechanisms and emotional processing. Therefore, it is possible that participants would be more able to upregulate control after trials with high cognitive demand during a neutral condition, where the distractive image is of neutral valence. This upregulation brought by the previous task’s cognitive demand would aid them in preventing the intrusion of an upcoming distracter stimulus. The unpleasant images, on the other hand, would be more prone to be captured automatically (Oliveira et al., 2013) and thus would consume processing resources that are also needed by cognition. Therefore, it would be more difficult in this case to prevent the intrusive processing of unpleasant distracters.

Our first aim was to use a WM paradigm to examine if adjustments of cognitive control that were based on previous trial load would interfere with the brain response to emotional and neutral distracters. We applied the experimental design from Figueira et al. (2017, 2018), which encompassed a change-detection WM task where each current WM trial task was preceded by a neutral or unpleasant image. Neural response to the image was assessed using the event-related potential (ERP) technique. Many ERP studies applying neutral and emotional images focus their analysis on an early time window (200–300 ms) after image onset at parieto-occipital electrodes. A common finding when comparing ERP responses to neutral and emotional images is a waveform component that tends to be more negative-going when evoked by the latter and has been denominated early posterior negativity (EPN; Schupp et al., 2003a,b). The more arousing the images are, the more negative-going are the EPNs computed for the individual images (Junghöfer et al., 2001; Wiens et al., 2011a,b), which leads to the conclusion that EPN presents an enhanced amplitude when evoked by motivationally salient stimuli (Schupp et al., 2003a,b, 2006). Besides the emotional content of the images, manipulations in attention seem to influence the waveform occurring 200–300 ms at the parieto-occipital electrodes after the image onset (De Cesarei et al., 2009; Wiens and Syrjänen, 2013). For instance, Wiens et al. (2011a) showed that the difference in the amplitude of the waveform for emotional as compared to neutral images in the 200- to 300-ms time window (i.e., the EPN) is eliminated when the images were task-irrelevant (i.e., when they are distracters). Interestingly, the absolute amplitude of the waveform evoked by both neutral and emotional images was also influenced by the task. The posterior waveform was more positive-going for both neutral and emotional images when they were task-irrelevant than task-relevant. Hence, this parieto-occipital waveform component 200–300 ms after the image onset for both low and high arousing stimuli seems to be influenced by whether the attention is directed or not to the stimuli (Nordström and Wiens, 2012; Wiens and Syrjänen, 2013). This waveform component, therefore, seems not only to be useful to track possible changes in the relative negativity evoked by emotional vs. neutral pictures but also may provide a measure of the allocation of attention to both neutral and emotional images (Wiens et al., 2011a).

It is important to note, however, that in many studies applying neutral and emotional pictures, the parieto-occipital waveform component at 200–300 ms after stimuli onset occurs superposed in the same time window and electrodes as a posterior P2 (Freunberger et al., 2007; Wiens et al., 2011b; Nordström and Wiens, 2012; Schupp et al., 2012; Todd et al., 2012; Wiens and Syrjänen, 2013). The posterior P2 evoked by non-emotional stimuli seems to reflect the electrophysiological correlate of the stimuli’s saliency (i.e., the perceived difference between the figure and its background), in a way in which the amplitude of the posterior P2 declines (become less positive-going) when the saliency increases (Straube and Fahle, 2010). The posterior P2 component is also enhanced by high cognitive demands. WM tasks have been shown to elicit modulations in the P2 waveform (Chapman et al., 1978; Dunn et al., 1998; Wolach and Pratt, 2001; Lefebvre et al., 2005; Finnigan et al., 2011), and a reduction in posterior P2 amplitude is related to a decrement in WM encoding (Finnigan et al., 2011). Moreover, during a high-load condition in a change detection task, the amplitude of the posterior P2 increases (Zhou and Thomas, 2015), suggesting a greater engagement of WM processes. The amplitude of posterior P2 for neutral images may also be influenced by the cognitive demands of the task. Freunberger et al. (2007) observed that neutral images evoked a greater posterior P2 amplitude during contexts with high cognitive processing demands than low cognitive processing demands.

Based on previous findings (Padmala et al., 2011) suggesting that adjustments in cognitive control occur when neutral images (non-emotional distracters) are presented, we hypothesized that the amplitude of the posterior waveform at the 200- to 300-ms time window after neutral image onset would be influenced by the load of the previous WM task. Based on the findings that there are shared resources between proactive control mechanisms and emotional processing (Padmala et al., 2011), the amplitude of this waveform component evoked by unpleasant distracter images would not be affected by adjustments in cognitive control that are imposed by a previous-trial load.

A second aim was to investigate whether self-reported thought control ability (Luciano et al., 2005) would influence the effect of the previous trial’s cognitive control adjustments on the neural response to the subsequent distracter. Because individuals with high thought control ability are well-succeeded in maintaining WM functioning optimal even in the face of unpleasant distracters (Figueira et al., 2017), we hypothesize that they will be more prone to apply cognitive control strategies, such as an upregulation that is based on previous trial WM load, in order to deal with distracters and shield goal-relevant information. The number of items that an individual can hold in WM (the individual WM capacity) has been previously associated with the capacity to control distracter access to WM (Vogel et al., 2005). Therefore, we also evaluated if thought control ability is associated with individual visual WM capacity.

## Materials and Methods

### Participants

The sample consisted of the same dataset of 36 participants (undergraduate students) from Figueira et al. (2018). From these, two were excluded due to excessive behavioral errors. The remaining 34 participants [22 women, mean (M) age of 21.88, standard deviation (SD) = 4.91] were used for behavioral analysis (WM capacity index or K index). For the electrophysiological analysis, three participants were excluded due to extensive eye movements and two due to extremely noisy data. The electrophysiological dataset of the remaining 29 participants (19 women, M_age_ of 21.67 years, *SD* = 4.69) were considered for the present study. All participants were right-handed (according to Oldfield, 1971), reported normal color vision and normal or corrected-to-normal visual acuity, had no history of psychiatric or neurological problems, and were free from any medication that acts on the central nervous system.

All participants gave informed consent before any experimental procedure was conducted. The participants did not receive any payment or reward to participate in the experiment as stipulated by the local ethics committee. The study protocol was approved by the ethics committee of Federal Fluminense University.

### Psychometric Evaluation (Thought Control Ability Trait)

The Thought Control Ability Questionnaire (TCAQ; Luciano et al., 2005) was used to assess the individual ability to control intrusive thoughts. The TCAQ is a self-report measure of an individual’s perceived ability to control unwanted, intrusive thoughts. The inability to control unwanted thoughts is present in a range of psychopathologies (Brewin et al., 2010). The TCAQ is a very well-known and widely used psychometric measure. It has been applied in research carried out in culturally diverse populations and in clinical psychology, among other fields (for review, see Feliu-Soler et al., 2019). This questionnaire comprises 25 items that are scored by the participant using a 1–5 scale based on their perceived ability to control intrusive thoughts. The total score can vary from 25 to 125, with higher scores reflecting higher thought control abilities. For the correlation analysis, we considered the TCAQ scores to be a continuum for individual variability in thought control. Based on previous findings (Figueira et al., 2017), we expected that participants would be equally able to upregulate control after high demanding trials (relative to low demanding trials) when neutral distracters are presented. However, when the distracters are unpleasant, the ability of participants to apply upregulation control based on previous trial WM load may vary as a function of their thought control ability.

### Experimental Stimuli and Procedure

#### Apparatus

The apparatus and stimuli used were the same as in Figueira et al. (2017). The experiment was conducted in a sound-attenuated room under dim ambient light. Eprime v2.0 software (E-Prime^®^ software-Psychology Software Tools Inc., Pittsburgh, PA, USA) was used to control stimulus timing and presentation to collect the responses. The participants positioned themselves on a head-and-chin rest that was 47 cm from the screen.

#### The Distractive Images

The 120 distractive images (20° × 16°) used in this study were equally distributed in two categories: neutral (intact bodies) and unpleasant (mutilated bodies) and were presented in a blocked fashion. The images were acquired from the International Affective Picture System (IAPS; Lang et al., 2008) and from the worldwide web. Images taken from the web were previously assessed in terms of hedonic valence and arousal following the protocol that was previously established by Lang et al. (2008) and Lang and Greenwald (1988). The images from the web were assessed on a scale of 1–9 in terms of valence (from unpleasant to positive) and arousal (from low to high) by a separate group of graduate students (*n* = 20) with ages similar to the current subjects (mean = 22.3 years old, *SD* = 1.81). In addition to the images of interest, we included different IAPS pictures as backgrounds, varying in valence and arousal, that served as the affective bases of comparison during the evaluation of the images of interest, thus anchoring the emotional rating scales. Unpleasant and neutral images differed in valence and arousal ratings (unpleasant images were more negative and arousing than neutral), as described in Figueira et al. (2017).

We also evaluated image complexity (i.e., simple figure-ground compositions vs. complex scenes), as early components are usually influenced by this aspect (Bradley et al., 2007; Wiens et al., 2011b). We employed a measure of compressed file size to extract the complexity of images (Marin and Leder, 2013). Unpleasant images showed significantly greater complexity than neutral images (*t*_(59)_ = 8.20; *p* < 0.001). The neutral and unpleasant pictures did not differ with regards to brightness, contrast, or spatial frequency (see Figueira et al., 2017). To assess whether the image’s complexity would affect the amplitude of the waveform in our study, we obtained mean amplitudes for each individual picture as previously suggested (Wiens et al., 2011b). The mean waveform amplitude was computed by averaging the ERP data across the relevant parieto-occipital electrodes and interval (200- to 300-ms time window) after the onset of each image. The mean amplitudes for each participant were averaged across individual images (rather than participants). We then conducted a Spearman correlation between the mean waveform amplitude and the complexity of each image. The correlation was significant (Rho = 0.40, *p* < 0.001), indicating that the image’s complexity would confound unpleasant vs. neutral comparisons.

#### The WM Task

The WM task used in this study was the change detection task (Vogel and Machizawa, 2004; Figueira et al., 2017, 2018). In the change detection task, the stimulus array was composed of two (low-load condition) or four (high-load condition) colored squares that were presented in both hemifields, which could display the following colors: white (RGB values: 255, 255, 255), black (0, 0, 0), red (255, 0, 0), blue (0, 0, 255), yellow (255, 255, 0), green (0, 255, 0), and purple (72, 61, 139).

The task began with the onset of an arrow cue for 200 ms, which signaled the hemifield to which the participant should covertly shift their attention. The arrow pointed to the left on half of the trials. The arrow cue was then replaced with a memory array that remained on the screen for 100 ms. After a 900-ms retention interval (RI), a test array followed and remained on the screen until the participant’s response at a maximum of 2,000 ms. Participants compared the test array to the previously shown memory array and responded if a square on the previously cued hemifield changed color or not by pressing one of the two buttons with the index finger from their right or left hand (Choice Reaction Time). The task had two difficulty levels: a low-load easier level in which the array consisted of two squares, and a high-load difficult level in which the array consisted of four squares. “Change” and “no change” responses were equally distributed within the high-load (four squares) and within the low-load (two squares) conditions. Therefore, high-load and low-load conditions were performed by left or right hand. The colors and spatial positions of the squares also varied throughout the trials to avoid priming effects (Ullsperger et al., 2005). Which response hand (left or right) was related to “change” and “no change” responses was counterbalanced across participants. The test array changed in comparison to the memory array in half of the trials. The next trial was initiated immediately after the response of the participant. Participants were instructed to respond as quickly as possible and to try not to commit errors. For more details about the WM task performed, see Figueira et al. (2017).

#### The Experimental Session

The experimental session consisted of four blocks, with 60 trials in each block. In half of the blocks (two blocks), a neutral image preceded the WM task during each trial, while in the remaining blocks, the WM task was preceded by an unpleasant image. The image was not relevant for performing the WM task (i.e., it was a distracter). The block order was counterbalanced across participants. Participants would take about 7 min per block, with about a 5-min interval between blocks to rest.

**Figure 1:**
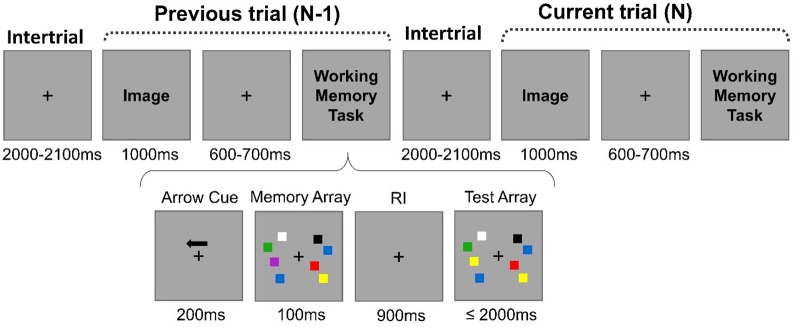
Example of the sequential order of events in two subsequent trials. Depending on the block, the image was neutral or unpleasant. The arrays of the working memory (WM) task could be comprised of four squares (high-load condition) or two squares (low-load condition). During the WM task, participants were instructed to respond whether one of the squares on the cued hemifield changed color by comparing the test array with the previously shown memory array. The image was always irrelevant to the task. The trial N-1 was followed by the trial N after an intertrial interval of 2,000–2,100 ms.

The entire sequential order of events in two subsequent trials is illustrated in Figure 1. Each block of trials initiated with the presentation of a fixation cross centrally localized that would stay in the screen throughout the entire experiment. Each trial N-1 ended with the WM task (change detection task; Vogel and Machizawa, 2004). Participants executed the WM task under low (two squares) or high (four squares) WM load conditions. After the stimuli presented in the WM task during the trial N-1 were turned off, only the fixation cross remained on the screen during 2,000–2,100 ms (intertrial interval). Then, a distractive image that could be neutral or unpleasant, depending on the block condition, would appear centrally on the screen. None of the distractive images were repeated across trials within a block. The image would stay on the screen for 1,000 ms. From 600 to 700 ms after the image offset, the WM task would begin again (trial N). The trials were balanced in terms of the previous- and current-trial load levels (i.e., high-load preceded by high-load trials; high-load preceded by low-load; low-load preceded by low-load; and low-load preceded by high-load).

### Electroencephalogram (EEG) Recording and Preprocessing

The electroencephalogram (EEG) data were recorded continuously from 64 active electrodes placed in an electrode cap (BrainProducts, Munich, Germany) according to the International 10-20 System. All data were recorded using a 500-Hz sampling rate, with the online reference set to the Cz and grounded at the FPz electrode. The impedance across all electrodes and experimental sessions did not exceed 20 kΩ.

The data were preprocessed in accordance with Keil et al. (2014) guidelines using BrainVision Analyzer 2.0 software (Brain Products, Munich, Germany). The data were band-pass-filtered between 0.01 and 30 Hz (24 dB/octave roll-off). We removed the eye-blink artifact using the available independent component analysis (ICA) tool in BrainVision Analyzer 2.0 software. The detection and reduction (maximal of two) of these components were made only after visual inspection of topographical maps (Jung et al., 2000). Topographical maps should demonstrate their proximity to the ocular area and established waveform characteristics.

Regarding the preprocessing of the waveform, the data were re-referenced to an average reference of all electrodes, as recommended (Hajcak et al., 2012; Liang et al., 2017). The epochs were set to 1,200 ms, starting 200 ms before the onset of the distractive image and ending 1,000 ms later. The baseline correction was performed at the −200- to 0-ms interval relative to the stimulus onset. The epochs were automatically detected for possible rejection based on the absolute voltage criterion (±100 μV). The validity of this automatic procedure was further tested by visually inspecting the data before definitive rejection. The epochs were averaged under consideration of the different image conditions (neutral or unpleasant) and the previous task load (N-1; two squares or four squares). The mean peak amplitude of the electrodes P7, P8, PO7, PO8, O1, Oz, and O2 was extracted over a 200- to 300-ms time window for the ERP statistical analysis.

The parieto-occipital waveform obtained at the 200- to 300-ms time window after image onset was used to uncover the previous trial WM task load effect on the neural response to the image. Therefore, epochs following trials with incorrect responses to the WM task (trial N-1) were excluded. The average epoch rejection rate was 25%.

### Statistical Analysis

We aimed to test if cognitive control adjustments that were based on the previous trial (N-1) would interfere with the neural response to the distractive image (neutral or unpleasant) taking into account the individual variability. The direct comparison between the neutral and the unpleasant conditions were not our main focus; thus, the analyses for “neutral-distracter” and the “unpleasant-distracter” blocks were completed separately. Also, differences in complexity that were related to neutral and unpleasant images prevented the direct comparison of neutral and unpleasant conditions because early visual ERPs are more vulnerable to differences in images’ complexity (Bradley et al., 2007).

For all analyses, the *p*-value considered for significance was *p* ≤ 0.05.

#### The Parieto-Occipital Waveform (200–300 ms)

To examine if the previous trial load (N-1) affected the neural response to the distractive image, we compared the parieto-occipital waveform obtained when the previous trial was a low-load trial (N-1; two squares) with the waveform obtained when the previous trial was a high-load trial (N-1; four squares). Nonparametric Wilcoxon tests were performed (separately for neutral and unpleasant conditions) because ERP data violated the assumption of normal distribution as assessed by the Shapiro–Wilk *W*-test (neutral: *W* = 0.88, *p* ≤ 0.05; unpleasant: *W* = 0.88, *p* ≤ 0.05).

Differences in accuracy during low-load (two squares) and high-load (four squares) conditions are expected. Indeed, in our task, the participants were more accurate in the two squares condition than the four squares condition (neutral: *t*_(28)_ = 11.04, *p* < 0.001; unpleasant: *t*_(28)_ = 13.94, *p* < 0.001). ERP waveforms are derived from averaged epochs and ERP amplitudes may change as the number of trials averaged increases (Thomas et al., 2004). Only the epochs in which participants performed correctly during the previous (N-1) WM task trials were included to compute this ERP waveform. Therefore, it would be possible for the number of epochs to be averaged to interfere in the amplitude of the waveform during the low-load (two squares) and high-load (four squares) conditions. To investigate this possibility, we performed a Spearman correlation between participants’ overall accuracy (considering all the conditions) and the parieto-occipital waveform’s amplitude to test whether the number of trials to be averaged would correlate with the amplitude of the ERP waveform in our sample.

#### Thought Control Ability and the Parieto-Occipital Waveform

We conducted a Spearman’s rank correlation test to determine whether the level of self-reported thought control ability (TCAQ scores) was related to the effects of the previous trial (N-1) load on the parieto-occipital waveform amplitude. To create an index that showed this ERP’s modulation by previous task load demand (named ERP by load demand index), we subtracted the mean amplitude of the waveform (at the 200- to 300-ms time window and parieto-occipital electrodes) evoked when the images were preceded by a low-load WM task (two squares) from the waveform activity evoked when the images were preceded by a high-load WM task (four squares).

Two separate correlations applying the TCAQ scores and the ERP by load demand index as variables were conducted: one for the neutral condition and another for the unpleasant condition.

#### Thought Control Ability and WM Capacity (K Index)

To evaluate if the thought control ability trait is associated with individual WM capacity, we calculated the K index, which measures the behaviorally transient WM capacity that is based on the ratio of correct and incorrect responses on a change-detection task. Based on the WM task, we estimated the behavioral index of visual WM capacity using Pashler’s formula: K = Sx(H-FA)/(1-FA; Pashler, 1988; Rouder et al., 2011), where K is the number of items maintained in WM, S is the array size (two or four squares), H is the hit rate, and FA is the false alarm rate. The K index of WM capacity is traditionally obtained during change detection tasks without the presence of emotionally laden stimuli (e.g., Dai et al., 2019). Therefore, we averaged the K indexes across the four and two squares conditions during the neutral trials to obtain an individual index of the WM capacity (Dai et al., 2019). We considered current trials (N) preceded by correct trials (N-1) to compute the K index, as applied for the electrophysiological data. We conducted a Spearman’s rank correlation between the K index and the TCAQ scores to test a possible relationship between the WM capacity and the thought control ability.

## Results

### Effect of Previous Trial (N-1) Load Level on the Neural Response to the Subsequent Image (Distracter)

**Figure 2:**
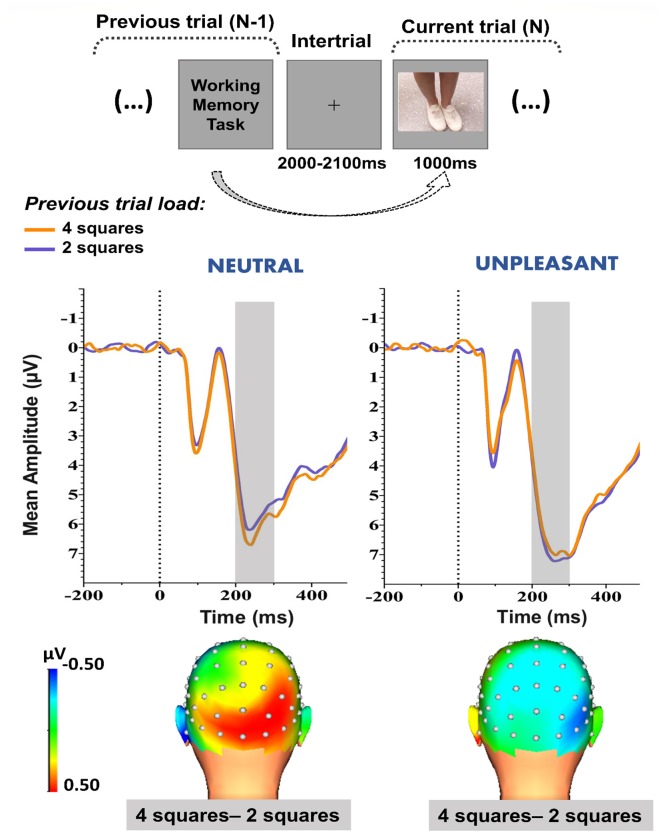
Upper panel: Schematic representation of the proposed method to evaluate the effects of the WM task’s previous trial demand on the images’ neural response. Middle panel: Early (200–300 ms) grand-average waveforms (collapsed across P7, P8, PO7, PO8, O1, Oz, and O2 electrodes) obtained during the high-load (four squares) and low-load (two squares) previous trial conditions. The shaded area represents the time window applied for analysis. Lower panel: 3-D topographic maps of the differences in the waveform mean amplitudes (200–300 ms) between high-load and low-load previous trial conditions. Neutral and unpleasant condition waveforms and topographic maps are depicted on left and right panels, respectively.

As shown in Figure 2, the parieto-occipital waveform presented a positive polarity over the parieto-occipital sites. Three-dimensional (3-D) topographic maps of the mean voltage amplitudes in the 200- to 300-ms time window (Figure 2, lower panel) revealed a more positive-going waveform (i.e., enhanced positivity) at the parieto-occipital sites when the distractive images were preceded by a high-load (four squares) WM task than a low-load (two squares) WM task during the NEUTRAL condition (Figure 2, lower panel, on the left). This activity pattern, as a function of previous trial load, was not observed during the UNPLEASANT condition (Figure 2, lower panel, on the right).

For the NEUTRAL condition, the Wilcoxon test showed that the waveform’s amplitude was greater (i.e., more positive-going) when the previous trial (N-1) consisted of four squares, as opposed to two squares (Figure 2, middle panel, on the left), at the 200- to 300-ms time window [*Z* = 2.28, *p* ≤ 0.05; Median (Med)_2 squares_ = 4.49 μV; Med_4 squares_ = 5.54 μV]. Therefore, it is possible to say that the previous trial load (N-1) affected the neural response to the subsequent distractive neutral image.

For the UNPLEASANT condition, there was no effect caused by the previous trial (N-1; *Z* = 0.66, *p* = 0.51; Med_2 squares_ = 6.69 μV, *SD* = 3.8; Med_4 squares_ = 5.31 μV, *SD* = 4), meaning that the emotionality conferred by the unpleasant image might have interfered with the previous trial load (N-1) effect.

The overall accuracy did not correlate with the parieto-occipital waveform amplitude (neutral: Rho = 0.09, *p* = 0.51; unpleasant: Rho = 0.04, *p* = 0.75), which suggests that the waveform’s amplitude results were not influenced by the number of trials to be averaged.

### Thought Control Ability Trait and Distracter Processing as a Function of the Previous Trial WM Load

**Figure 3:**
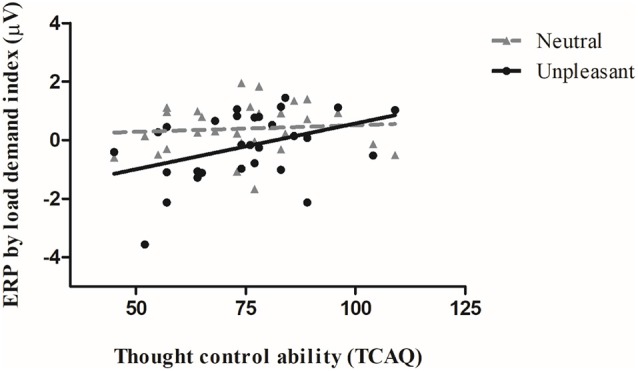
Correlation between the participants’ mean event-related potential (ERP) load demand index amplitude (μV) and the thought control ability trait scores during the unpleasant (black filled line and circles) and neutral (gray dashed lines and triangles) conditions. The greater the ERP by load demand index, the greater the difference between the high-load and low-load WM tasks (N-1).

We observed a significant Spearman correlation between the ERP by load demand index and the thought control ability trait during the UNPLEASANT condition (Rho = 0.40, *p* ≤ 0.05). The correlation was not significant for the NEUTRAL condition (Rho = 0.14, *p* = 0.46). Both correlations can be seen in Figure 3. The greater the “ERP by load demand index,” the greater the parieto-occipital waveform amplitude evoked when the image was presented after a high-load condition relative to a low-load condition. Therefore, for the UNPLEASANT condition, we found that the greater the thought control ability, the greater the ERP by load demand index.

### Thought Control Ability and WM Capacity (K Index)

The thought control ability trait was positively associated with individual WM capacity. The higher the WM capacity (K index), the greater the thought control ability (TCAQ scores; Rho = 0.34; *p* ≤ 0.05).

## Discussion

### Main Findings

The present study aimed to investigate whether cognitive control adjustments that were based on the previous trial (N-1) interfered with the neural response to distractive images (neutral or unpleasant), considering individual differences in thought control ability. The parieto-occipital waveform evoked by a neutral distractive image was more positive-going when the image was preceded (trial N-1) by a high-load WM task than a low-load WM task. The load level of the previous (N-1) WM task did not interfere with the processing of the subsequent image when it was unpleasant. During the unpleasant condition, there was a correlation between the level of self-reported thought control ability and the effects of the previous trial (N-1) load on the parieto-occipital waveform’s amplitude (ERP by load demand index). The lower the thought control ability, the less positive-going was the parieto-occipital waveform evoked by the unpleasant image when it was preceded by a high-load (vs. low-load) WM task. In line with these findings, the thought control ability trait was also positively associated with WM capacity.

### The Parieto-Occipital Waveform

The parieto-occipital waveform (at the 200- to 300-ms time window) reported here occurs in a time window and topography shared by two distinct ERPs, the EPN and the posterior P2. The former features a relative increase in negativity for emotional stimuli (Schupp et al., 2003a,b, 2006), besides possibly indexing the allocation of attention (Wiens et al., 2011a), whereas the posterior P2 is a positivity reported to be influenced by executive cognitive processing (Freunberger et al., 2007; Finnigan et al., 2011; Zhou and Thomas, 2015; Gouet et al., 2018). Considering that in the present study we were unable to directly compare the ERP elicited by neutral and unpleasant images, due to differences in image complexity, we should be cautious about interpreting our results in terms of an EPN. Therefore, we chose to interpret the parieto-occipital waveform as a posterior P2, which aligns our results in terms of adjustments in cognitive control with previous research evidencing a relationship between the posterior P2 and top-down activation (Freunberger et al., 2007).

### WM Load May Trigger Dynamic Adjustments in Cognitive Control and Influence Neural Response to Neutral Distracter

We demonstrated that mnemonic demands in a change detection task may trigger upregulation and affect the processing of a following non-emotional distracter. Maintaining task-relevant visual representations while avoiding task-irrelevant representations is a core function of WM (Repovs and Baddeley, 2006). WM is considered to play a role in cognitive adjustments through a sustained maintenance of goal-relevant stimuli features (Braver, 2012). De Pisapia and Braver (2006), for example, suggested that transient, trial-by-trial upregulation control may coexist with a proactive control strategy that is related to the sustained maintenance of the task set. It has been proposed that loading WM with visual-feature maintenance encoding, for example, by increasing the set size for a memory sample of colors, would increase the posterior P2 amplitude (Zhou and Thomas, 2015). It has also been reported that the posterior P2 is related to nonsymbolical numerical processing, with its amplitude increasing according to the effort necessary to discriminate between the numerical magnitude of arrays (Libertus et al., 2007; Hyde and Wood, 2011; Gouet et al., 2018; Liu et al., 2018). This is in agreement with evidence that the posterior P2 component is enhanced by high cognitive demands. The posterior P2 has also been implicated in the detection of deviant, nontarget stimuli and in feature matching processing (Freunberger et al., 2007; Akyürek et al., 2010). It is possible that a previous high-load WM task enhances cognitive processes that facilitate the discrimination between relevant and nonrelevant features, promoting the positive-going shift of the P2 during the distracter neutral stimuli window. In the low-load condition, this higher-order discrimination of relevancy in features would be reduced, as indexed by a less-positive-going P2, which would enhance the perceived salience of the distracter (Straube and Fahle, 2010).

Simon et al. (2016), in an event-related study, found that there was a decrease in early auditory processing (indexed by the N1 ERP component) in response to distracters when under high WM load. They suggested that increased WM load is associated with enhanced cognitive engagement, shielding WM from distracters. Therefore, it is possible that previous high-load WM trials triggered an additional cognitive engagement strategy that is reflected during the distractive image processing. However, WM load studies usually present the distracters, either during the encoding of the set or during the maintenance delay. In our study, it seems that participants optimally biased cognitive preparation ahead of a distractive event.

### Adjustments in Cognitive Control May be Reduced During an Unpleasant Condition

During the unpleasant condition, we did not find an influence of the previous trial load on the neural response for the distracter, as the difference in the posterior P2 amplitude between previous (N-1) high-load and low-load trials was absent. Many studies argue that unpleasant emotional stimuli have a prioritized capture of attention due to its importance for survival by signaling possible danger (Vuilleumier and Schwartz, 2001; Öhman et al., 2001; Erthal et al., 2005; Pereira et al., 2010; Fernandes et al., 2013; Deweese et al., 2016; Shackman et al., 2016). Because of that, threat-related distracters are able to gain unnecessary access to WM (Stout et al., 2013, 2018), which disrupts its functioning, especially when negative distracters are shown during the maintenance period (Dolcos and McCarthy, 2006; Anticevic et al., 2010). Therefore, it is likely that highly unpleasant emotional stimuli, like the mutilated-body images applied here, reduce the adjustments in cognitive control and promote biased cognition toward affective stimuli, regardless of the previous trial load level. This interpretation is supported by the study of Padmala et al. (2011), which found that behavioral adjustments following incongruent trials were decreased when participants were exposed to mutilated-body images.

An important distinction should be made between the stimulus-driven and state-dependent emotional effects on adjustments in cognitive control. Because the neutral or unpleasant images were presented in a blocked fashion, a neutral or unpleasant emotional state was also induced (Figueira et al., 2017). Therefore, our experimental design allowed possible emotional effects promoted by the presentation of the unpleasant distractive image transiently, as well as by an unpleasant emotional state, which was induced throughout the block (Figueira et al., 2017). Besides the stimulus-driven disruptive effect of unpleasant stimuli on cognitive control adjustments (Padmala et al., 2011), control adaptations seem to also be dependent on one’s emotional state. Some studies suggest that unpleasant affective states will prioritize conflict processing, which will strengthen cognitive control adjustments (e.g., van Steenbergen et al., 2010; Schuch and Pütz, 2018). Although we cannot discard the possible influences of emotional states on control adjustments in our study, we found electrophysiological evidence that the influence of the previous trial WM load on distracter processing is absent (i.e., there is no control adaptation) when the distracter presents unpleasant content.

### Individual Abilities in Controlling Thoughts May be Related to Cognitive Control Adjustment Effects During an Unpleasant Context

Participants seemed to be similarly able to upregulate control after high demanding trials when neutral distracters were presented. The participants’ ability to apply upregulation control that is based on previous trial WM load varied as a function of their thought control ability when the distracters were unpleasant. Electrophysiological findings support the hypothesis that during an unpleasant context, the higher the thought control ability, the greater the cognitive adjustments, based on previous trial demands. Figueira et al. (2017) demonstrated through the CDA index (Luria et al., 2016) that transient task-relevant WM representations were less affected by unpleasant images the greater the thought control ability of the participants. Here, we extended this finding by showing that thought control ability is related to WM capacity and may align thoughts and actions in accordance with proactive strategies in order to keep task-goal information in mind while shielding WM from unwanted representations. In fact, WM capacity has been related to an increased ability to maintain task goals (Vogel and Machizawa, 2004) while filtering out distracters (Vogel et al., 2005). This has also been linked to a greater ability to dynamically and adaptively adjust the level of cognitive control, which minimizes susceptibility to distracters (Weldon et al., 2013).

These findings have important practical implications. Unwanted intrusive unpleasant thoughts are present in a range of psychopathologies (Brewin et al., 2010) and cause distress and suffering. Thought control ability (measured through TCAQ) has been shown to be reduced in posttraumatic stress disorder (PTSD) patients (Catarino et al., 2015) and to be related to PTSD symptom severity (Valdez and Lilly, 2012), impulsivity (Gay et al., 2011), and anxiety (Luciano et al., 2005). The electrophysiological and behavioral biological markers of thought control ability (in an unpleasant context) that is uncovered here may eventually aid in the detection of vulnerability and resilience factors for the development of some psychopathologies, like PTSD and/or obsessive-compulsive disorders. This assumption is in line with the Research Domain Criteria (RDoC) initiative (Insel et al., 2010).

### Limitations

In our study, the complexity of the images was greater for the unpleasant stimuli relative to the neutral stimuli, which prevented us from investigating possible differences between unpleasant and neutral stimuli and discussing the data in terms of the EPN. Complexity affects the ERP at roughly the same interval and electrodes as the EPN (Bradley et al., 2007; Wiens et al., 2011b). These features might effectively mask the EPN, that is, the negative-going difference between unpleasant and neutral images (Van Strien et al., 2009). In fact, we did not observe this relative negativity in the posterior waveform in our study. In addition to the difference in complexity between the unpleasant and the neutral images, the images were task-irrelevant (distracters). Previous studies showed that the EPN is eliminated or reduced when images are task-irrelevant (Wiens et al., 2011a). Therefore, future studies should consider these factors when investigating the influence of previous trial load on the waveform difference between unpleasant and neutral images.

It is also worth mentioning that we did not include pictures with pleasant valence in our study. Trait-positive affect is related to better performance in the WM task applied in the present study (Figueira et al., 2018). Because of the absence of pleasant stimuli in our study, we were unable to uncover the interplay between WM and positive emotions. It would be interesting to address this issue in the future.

## Conclusion

In summary, this study contributes to the understanding of how cognitive control mechanisms act to flexibly keep relevant information in mind while dealing with neutral or unpleasant distracters and support goal-directed behavior. Dynamic cognitive control adjustments are impaired by an unpleasant context. Individual abilities to control thoughts may mediate the effect of cognitive control adjustments on distracter processing in the presence of unpleasant environment cues. The ability to control unwanted, unpleasant thoughts is linked to individual differences in the ability to promote dynamic control adjustments over unpleasant distractive stimuli. These findings also characterize biological marker results and may have potential for practical implications.

## Data Availability Statement

The datasets generated for this study are available on request to the corresponding author.

## Ethics Statement

The studies involving human participants were reviewed and approved by Federal Fluminense University Hospital Ethics Committee (HU). The protocol was approved by the University Hospital Ethics Committee (HU, CAAE: 53505615.0.0000.5243). The patients/participants provided their written informed consent to participate in this study.

## Author Contributions

ID, MP, LO, JF, and LP developed the study concept and the study design. LP and JF performed the data collection. LP, JF, and ID performed the data analysis and interpretation. LP and ID drafted the manuscript. MP, LO, and JF substantially contributed to the interpretation of the data and provided important critical revisions. All authors approved the final version of the manuscript. They also agreed to be accountable for all aspects of the work.

## Conflict of Interest

The authors declare that the research was conducted in the absence of any commercial or financial relationships that could be construed as a potential conflict of interest.
